# A pilot study on feasibility, acceptance and effectiveness of metacognitive-oriented social skills training in schizophrenia

**DOI:** 10.1186/s12888-017-1378-z

**Published:** 2017-06-12

**Authors:** Felix Inchausti, Nancy V. García-Poveda, Alejandro Ballesteros-Prados, Eduardo Fonseca-Pedrero, Javier Ortuño-Sierra, Sergio Sánchez-Reales, Javier Prado-Abril, José Antonio Aldaz-Armendáriz, Joe Mole

**Affiliations:** 1 Complejo Hospitalario of Navarra, CSM Ermitagaña, and School of Medicine, University of Navarra, Pamplona, Spain; 2 Complejo Hospitalario of Navarra, CSM Ermitagaña, Pamplona, Spain; 3 Complejo Hospitalario of Navarra, CSM Estella, Estella, Spain; 4 Department of Educational Sciences, University of La Rioja, and P3 Prevention Program of Psychosis, Oviedo, Spain; 5 Department of Educational Sciences, University of La Rioja, Logroño, Spain; 6 Virgen del Castillo Hospital, CSM Jumilla, Murcia, Spain; 7 Complejo Hospitalario of Navarra, CSMIJ Natividad Zubieta, Sarriguren, and Research Network on Preventive Activities and Health Promotion (REDIAPP) (RD12/0005), Aragon Health Sciences Institute (IACS), Zaragoza, Spain; 8 CSM Sagasta, Miguel Servet University Hospital, Zaragoza, Spain; 9 Oxford Institute of Clinical Psychology Training, University of Oxford, Oxford, UK

**Keywords:** Metacognition-oriented social skills training (MOSST), Schizophrenia, Recovery, Social functioning, Metacognition

## Abstract

**Background:**

In preparation for a randomized controlled trial, a pilot study was conducted to investigate the feasibility, acceptability and effectiveness of a psychotherapy group based on metacognitive-oriented social skills training (MOSST).

**Methods:**

Twelve outpatients with schizophrenia were offered 16 group-sessions of MOSST. Effect sizes were calculated for changes from baseline to treatment end for both psychosocial functioning and metacognitive abilities measured by the Personal and Social Performance Scale (PSP) and the Metacognition Assessment Scale–Abbreviated (MAS–A) respectively.

**Results and discussion:**

Ten patients finished the full treatment protocol and nonsignificant moderate effect sizes were obtained on PSP and MAS–A scores. To date, this is the first study in Spain to suggest that outpatients with schizophrenia will accept metacognitive therapy for social skills training and evidence improvements in psychosocial functioning and metacognition.

**Conclusion:**

Despite limitations inherent in a pilot study, including a small sample size and the absence of a control group, sufficient evidence of effectiveness was found to warrant further investigation.

**Trial registration:**

ISRCTN10917911. Retrospectively registered 30 November 2016.

## Background

Although not included in the diagnostic criteria, impaired social functioning is considered one of the most common features of schizophrenia spectrum disorders and has been widely described in the literature [1]. Examples of this set of deficits include poor management of conflicts, difficulty conversing, and aggressive behavior towards family, friends, community members and/or co-workers. As a result, social skills training (SST) has emerged as a well-validated intervention that is recommended in several treatment guidelines for schizophrenia [2, 3]. However, many studies have found that the effects of these interventions on patients’ social functioning, and the generalizability of these effects, are poorer than desired. For instance, a meta-analysis conducted by Pilling et al. [4] did not find any significant benefit of SST clinical trials. Similarly, Kurtz and Mueser [5] found that SST produced only a modest effect size with respect to improvements in psychosocial functioning (*d* = 0.52), and there was only a small effect size for relapse reduction (*d* = 0.23). Thus, a recent Cochrane Collaboration Review [3] has concluded that, to date, it remains unclear whether current SST programs are more effective than standard care.

This literature has spurred efforts to increase the impact of SST interventions for people with schizophrenia spectrum disorders [6–8]. One promising approach is based on the hypothesis that metacognitive deficits are the key barrier to adaptive social functioning in psychosis [9]. Within a broad term of metacognition, it refers to the range of mental activities that allow people to be aware of and reflecting upon their own thoughts, feelings, and intentions, and those of other people, and ultimately formulate connections between these events into larger complex representations of themselves and others [10]. Metacognitive deficits may vary from those that affect the ability to differentiate reality from fantasy to the capacity to empathize with others, and to think flexibly about mental states. In the literature, these skills have also been referred to as social cognition, theory of mind, or mentalization, to name but a few. In this work, the construct of metacognition is used as an “umbrella” term for all the mental processes that underlie social interaction. A wealth of evidence shows that individuals with schizophrenia have deficits on more discrete facets of metacognition such as the perception of emotional information [11], as well as more synthetic aspects of metacognition, such as the integration of information into larger representations of oneself, others and the world [12].

It may be logical to assume that adaptive social functioning requires understanding of one’s own and other’s mental states. Thus, effective social performance demands self-reflectivity, care about others, and the management of emotional arousal that frequently follows intersubjective experience. To understand other people also requires one to imagine being in a similar situation to others, share the feelings of others, which goes beyond just guessing his/her emotion, and then manage any resulting feelings [13]. Given the importance of these skills, remediating metacognition may be an imperative component of effective SST. Unfortunately, many existing SST almost entirely neglect the promotion of the metacognitive abilities [14]. In light of this, Ottavi et al. [15] have recently developed a novel metacognition-oriented SST (MOSST) intervention that includes metacognitive remediation as a means of improving the effectiveness of conventional SST for patients affected by psychosis. MOSST is a structured manualized intervention designed to stimulate participants’ capacity to reflect on the thoughts, emotions, and intentions of others [16]. To date, MOSST has been used with small groups of patients with long-term schizophrenia on partial hospitalization or with first-episode psychosis [16]. As far as we know, the only published study on the effects of MOSST is a case illustration wherein a 56-year-old individual with schizophrenia successfully completed the full program [15]. After intervention, the authors reported improvements in the patient’s ability to be aware of, understand and communicate his own and other people’s mental states, and to evoke adaptive social behavior during role-play. The skills that this patient acquired during training also widely generalized to the ‘real-world’, which had a largely positive impact on his quality of life. However, there is still a lack of empirical support for these observations.

As a precursor to a RCT for MOSST (trial registry as ISRCTN10917911), we conducted a pilot study to clarify previous aspects. In particular, we sought to investigate (1) whether new therapists could be trained in MOSST, and the level of post-training supervision that would be necessary. Secondary data were collected to (2) estimate the magnitude of the intervention effect and therefore determine the required sample size for a RCT, (3) verify the acceptance rate of the therapy, and (4) determine whether the intended test battery and its administration was feasible.

## Methods

### Therapists and training

To determine the feasibility of training therapists in MOSST, two Spanish therapists (NG and AB) who had over 4 years’ experience conducting groups and SST, were trained by the author of the Spanish treatment protocol (FI) in a 4-day training program. Training consisted of 1 day of theoretical work, focusing on the construct of metacognition and the use of the Metacognition Assessment Scale–Abbreviated (MAS–A) in psychosis [10, 17]. This knowledge was tested in the second day during a MAS–A consensus meeting using “gold standard” transcriptions specifically developed for the training, which are included in both the English MAS–A manual and the Spanish translation. The remaining 2 days involved expansive training on core elements of MOSST. Training mainly focused on developing the therapists’ capacity to act as metacognitive facilitators. This included methods for helping participants to (i) produce significant narrative episodes, (ii) deduce mentalistic elements from these episodes, and (iii) provide clear metacognitive feedback and self-disclosures during role-play exercises. Basic caseworks were also discussed. In order to promote metacognition, therapists were encouraged to use five specific therapeutic skills. Firstly, therapists should adopt a validating attitude. Secondly, therapists should use transparent, honest, clear and simple communication, as well as well-structured comments in order to stimulate patients’ capacity to understand the other’s mind, as opposed to opaque, figurative, and evocative expressions. Thirdly, each intervention should be as focused as possible on metacognitive contents. An illustration of this skill taken from Ottavi et al. [16] could be, for example, when a patient requests to leave the group session to smoke whilst another group member is making an important revelation. In such situations, the therapist could respond: “I think X and I might get offended or upset if you leave the group now. I would like to finish the session with all of us together, and I would like to hear your observations about what X is telling us. I would also feel concern about not being able to keep the group together and motivate you all” (p. 297). Fourthly, therapists should normalize patient concerns to generate a sense of sharing [18]. Fifthly, there should be intensive use of metacommunication including self-disclosing and self-involving statements in therapist-patient interactions [19, 20].

Sufficient learning of the therapy method was assessed by performance during role-play exercises. Two therapists (NG and AB) conducted therapy sessions under the supervision of FI throughout the study.

### Therapy protocol: MOSST

MOSST operates according to a hierarchical model of metacognition in schizophrenia where people must be able to perform relatively less complex metacognitive tasks (e.g., knowing their thoughts are their own) before being able to perform more complex ones (e.g., knowing how thoughts and feelings are connected to daily life). As in successful metacognitive-focused psychotherapy, different metacognitive abilities are supposed to develop before others, with improvements in self-reflection occurring before the capacity to use metacognitive knowledge to respond to psychological and social challenges [17].

MOSST has been developed for in- and/or outpatients affected by schizophrenia spectrum disorders, and the full protocol is based on evidence suggesting that difficulties in metacognition are partially dependent on problems in the intersubjective domain, rather than abilities that can be taught [21]. It is generally recommended that MOSST groups should be composed of 5 to 10 participants in order to be large enough to be stimulating and produce an atmosphere of cooperativeness among group members, but not too large as to be chaotic or marginalizing for more introverted participants. The Spanish protocol involves 16 weekly group-sessions lasting approximately 90 min each, in which 16 social skills are trained according to a criterion of increasing difficulty. The duration of the treatment was determined for several reasons. Firstly, by the number of social skills that MOSST intends to train; the structure of group sessions makes it difficult to train more than one social skill per session in this patient profile. Secondly, because it has been observed that a minimum period of 4 months is necessary for metacognitive capacity growth during therapy [22].

The target social skills have been divided into: (i) conversation skills such as listening to others, greeting others, starting and ending conversations, and maintaining conversations; (ii) assertiveness skills such as making and refusing requests, making and receiving compliments, asking for information, suggesting activities to other people, and expressing unpleasant and positive feelings; and (iii) conflict management skills such as compromise and negotiation, making productive complaints, responding to negative complaints, and making apologies [16]. MOSST incorporates some essential elements of standard SST such as role-play exercises, but seeks to expand their application by stimulating metacognitive activity and discussion of the therapeutic relationship as it occurs in the moment. There are several main features of MOSST: (i) the attention to metacognitive abilities which makes MOSST more than a teaching experience or an exercise in changing dysfunctional interpersonal thoughts; (ii) the promotion of rich and articulated understandings of one’s own mental states and those of others, to allow participants to act more effectively in social contexts; (iii) the focus on intersubjectivity as a way to enhance thinking about the relationships in which patients and therapists are emotionally involved; and (iv) the use of the metacommunication to express openly the mental states of oneself and the others during role-play exercises.

In sum, MOSST may offer a first-line therapeutic intervention focused on self-reflection and understanding the mind of the others.

### Participants

In order to answer research question 2 regarding clinical gains, so as to inform the sample size required for a RCT, and research question 3, pertaining to the acceptance rate of the therapy, 12 participants were recruited from two mental healthcare services in Navarra, a region located in the north of Spain. Because this is a time consuming intervention protocol with people with a severe mental disorder, recruitment was carried out assuming a drop-out rate of 15%. The purpose was for a minimum of 10 patients completed the full program.

Candidates aged 18–65 years were receiving routine treatment and either met International Classification of Diseases–tenth revision (ICD–10) criteria for schizophrenia, schizoaffective disorder, or delusional disorder, as determined by trained Psychiatrists or Clinical Psychologists through clinical interview. All participants were clinically stabilized and treated with a stable dose of the same antipsychotic for at least 2 months. Participants demonstrated social engagement problems and poor participation in social activities via case manager reports. Participants were excluded if they were non-Spanish-speaking or had concomitant substance abuse, moderate to severe learning disabilities or developmental disorders, major neurological illness, impaired intellectual functioning (Wechsler Adult Intelligence Scale–IV Full Scale IQ score < 70), or did not have the capacity to consent to research participation.

The case managers of eligible patients were also consulted about whether each case might be suitable for inclusion in the study, when their individualized treatment plan was considered. Following this, suitable patients were invited to participate. The sample was predominantly male (*n* = 10), with a mean age of 36.40 years (*SD* = 11.60), a median level of secondary education, and an average estimated premorbid IQ of 90.40 (*SD* = 5.63). Baseline values for the Positive and Negative Syndrome Scale (PANSS) scores are provided in Table 1 to allow comparison to other patient samples. All participants were part of the same treatment group and they received no type of incentive for taking part in the study.

**Table 1:** Sample characteristics on sociodemographic and clinical variables at baseline

	*f* or Mean (*SD*) *N* = 12
Gender	
Males	10
Females	2
Age	36.40 (11.60)
WAIS–IV Full Scale IQ	90.40 (5.63)
PANSS	
Positive	14.58 (4.12)
Negative	20.16 (9.24)
Global	37.65 (11.48)
Total	72.39 (23.29)

*f* frequency; *SD* standard deviation; *WAIS*–*IV* Wechsler Adult Intelligence Scale–IV; *PANSS* Positive and Negative Syndrome Scale

### Measures

Psychosocial functioning and metacognition were assessed using the Personal and Social Performance Scale (PSP) [23] and the MAS–A [10, 17], respectively, to measure clinical gains, as per research question 2. The PSP is a clinician-rated instrument that evaluates the functioning of patients in four areas independent of symptomatology: self-care, socially useful activities, personal and social relationships, and disturbing and aggressive behaviors. Each of these four domains is assessed by an item rated between 0 (absent) and 5 (very severe); lower scores indicate better functioning in the domain. The PSP also provides a total score between 1 and 100; higher scores represent better personal and social functioning. The timeframe used was the level of performance in the last month. The Spanish PSP has proved to be a reliable, with a Cronbach’s alpha of 0.87, valid and sensitive instrument for the assessment of social functioning in patients with schizophrenia [23]. In this study, the Cronbach’s alpha coefficients of the PSP scores were 0.81 at pre-test and 0.83 at post-test. The MAS–A is a rating scale for assessing different forms of metacognitive activity within personal narratives. It contains four subscales: ‘Self-reflectivity’ assesses the comprehension of one’s own mental states; ‘Understanding the Other’s Mind’ considers the comprehension of other individuals’ mental states; ‘Decentration’ evaluates the ability to perceive the world as existing with others having independent motives; and ‘Mastery’ assesses the ability to use knowledge of one’s mental states to define psychological problems and adequately deal with them. The MAS–A total score ranges from 0 to 28, and is generated by summing the scores of the four subscales. Higher scores on the subscales indicate higher capacity to integrate and effectively use intersubjective information. Available psychometric evidences indicate that the MAS–A has acceptable values of internal consistency, and test-retest and inter-judge reliability, with intraclass coefficients between 0.71 and 0.91 [24]. In this study, the inter-raters reliabilities of the MAS–A scores were 0.91 at pre-test and 0.90 at post-test. Participants’ narratives were obtained using the Spanish adaptation of the Metacognition Assessment Interview (MAI) [10]. The MAI is a 30–60 min-long semi-structured interview designed to elicit a vivid narrative about the most troubling interpersonal experience in the last 4 months. This timeframe was selected in order to facilitate recall and allow for test-retest assessment. All assessments were conducted before and after therapy by independent raters blind to condition (pre- or post-test). All raters had successfully completed a prior 4-h PSP and MAS–A training session delivered by FI, and subsequently attended three consensus meetings as part of the training. In addition, acceptability and subjective impact of the intervention were assessed by an anonymous self-report scale at the end of each session to evaluate the session’s enjoyableness, usefulness and effect on daily social functioning using a 5-point scale (1 = fully disagree, 2 = disagree, 3 = not sure, 4 = agree, 5 = fully agree) (see Table 2).

**Table 2:** List of feedback items after group sessions

Item	Content
1	Training was useful and sensible
2	I had to force myself to go to the training regularly
3	In every-day life, I do not apply the lessons learned
4	Training was an important part of my treatment program
5	I would have liked to spend the time doing something else
6	Training was fun
7	A lot of what I learned during training is useful to my daily routine
8	The goals and rationale of training were clear to me
9	I would recommend training to others
10	I found it beneficial that training was administered in a group

In line with our final question concerning the feasibility of the battery, additional secondary outcome measures were included. No analysis of these data will be conducted, however, given the limited sample size. Psychotic symptoms were assessed with the PANSS [25]; the effects of mood and anxiety were controlled with the Beck Depression Inventory–II (BDI–II) [26] and the Beck Anxiety Inventory (BAI) [27] respectively.

### Statistical analyses

Statistical analyses were performed using SAS (SAS Institute Inc., Cary, NC, USA). According to guidelines for pilot studies as specified by Arain et al. [28], data gathering was performed mainly to test the study design and gain clinical impressions of the methodology and process of the trial. As such, only effect size calculations (Cohen’s *d*) were performed on the PSP and MAS–A outcome measures. Results on secondary outcome measures are made available upon request.

## Results and discussion

This pilot study sought to examine the feasibility of a RCT to analyze the effects of a newly developed metacognitive SST: MOSST. Our first question was to determine whether new therapists could be trained in MOSST and what levels of post-training supervision are required. Both the supervisor and therapists felt that the method had been transferred successfully. Post-session meetings were helpful, both as a fidelity check and to guide therapists in identifying which elements of the therapy they had difficulty with and should be discussed in supervision. In relation to the levels of supervision required, therapists found that active participation in supervision was essential for successful application of MOSST. Although weekly supervision would be desirable, this may not be feasible in many public healthcare settings. A reasonable consensus between the supervisor and therapists was reached that fortnightly face-to-face supervision seems to be the minimum requirement (Table 3).

**Table 3:** Means (standard deviations) in relevant outcomes at pre- and post-treatment

	Pre-treatment *N* = 12	Post-treatment *N* = 10	*t*-value	*p*-value	*d*-value
PSP					
Self-care	2.00 (1.00)	2.00 (1.00)	0.00	1	0.00
Activities	2.67 (0.98)	1.65 (1.03)	1.02	0.12	1.01
Relationships	3.33 (0.49)	2.45 (0.60)	1.67	0.09	1.61
Behaviors	1.33 (0.49)	0.67 (0.99)	1.69	0.12	0.84
Total	53.31 (7.39)	60.00 (8.70)	−5,06	0.11	–0.83
MAS–A					
Self-reflectivity	3.67 (0.98)	4.33 (1.23)	−1.15	0.27	−0.59
Others	2.67 (0.98)	3.67 (1.10)	−1.59	0.14	−0.96
Decentration	1.33 (0.49)	1.83 (0.54)	−0.73	0.48	−0.44
Mastery	2.39 (0.68)	2.58 (0.71)	−0.45	0.66	−0.27
Total	10.06 (2.95)	12.41 (3.45)	−2.32	0.14	–0.73

*PSP* Personal and Social Performance Scale; *MAS*–*A* Metacognitive Assessment Scale–Abbreviated

Our second goal was to estimate the magnitude of clinical gains and determine the sample size required for a RCT. The following (non-statistically significant) effect sizes were obtained on PSP scores: self-care, 0; socially useful activities, 1.01; personal and social relationships, 1.61; and disturbing and aggressive behaviors, 0.84, (total, −0.83). Concerning MAS–A scores, effect sizes obtained were as follows: self-reflectivity, −0.59; understanding the other’s mind, −0.96; decentration, −0.44; and mastery, −0.27 (total − 0.73). Pre-test data from participants who dropped out did not affect the magnitude of these effect sizes. The effect size for both total PSP and MAS–A scores (−0.83 and −0.73 respectively) were entered in SAS. To detect such effects, or larger ones, using an independent samples *t*-test at the conservative alpha of 0.05 (two-tailed), a minimum sample size of 32 per group would be required. Assuming a 15% drop-out rate, a sample of 37 patients per group is needed.

It is worth highlighting the positive progress on psychosocial functioning of patients (*d* = −0.83), especially in relation to the increase of useful social activities (*d* = 1.01) as well as personal and interpersonal relationships (*d* = 1.61). The magnitude of these effect sizes was clearly larger than those reported in other studies analyzing the impact of standard SST. For instance, the meta-analysis conducted by Kurtz and Mueser [5] found only a medium effect size on psychosocial functioning (*d* = 0.52). Despite the small sample size of this pilot study, the results obtained with MOSST are encouraging to warrant further investigation.

Regarding change in metacognition, MOSST produced overall improvements on self-reflectivity (*d* = −0.59) and understanding the other’s mind (*d* = −0.96). Although some progress on decentration was also observed, these changes were weaker (*d* = −0.44). These findings revealed a pattern of metacognitive gains consistent with the previous case report on the effects of MOSST [15], as well as with results obtained in other metacognitively oriented psychotherapies [29, 30]. However, MOSST seems to be specifically aimed at improving self-reflectivity and the others’ understanding abilities. In previous studies gains in mastery were reported to have improved rather swiftly but in this study such gains were absent [22]. An explanation of this effect could be the fact that MOSST was primarily designed to increase the participants’ awareness of their own thoughts and emotions, and to enrich their perspective of others’ mental functioning. MOSST also seems to help patients to understand that their own thoughts are subjective experiences separate from the mind of others, and that their internal expectations do not have a direct effect on reality. Either way, our findings are consistent with a hierarchical model of metacognition in schizophrenia [17].

The large effect of MOSST on psychosocial functioning might also be explained in terms of metacognitive gains. If patients are more aware of their own mental states and those of others, they might, therefore, improve their thinking about the need for more friends and people who care about them, as well as of this need in others. Moreover, it seems important to note that expecting any change regarding social functioning requires improves the self-reflectivity (e.g., to become aware that people are closer) and the understanding of others’ mind (e.g., to develop plausible guesses about internal states of others). Given the limited sample size, no further interpretation of these data was ventured.

We thirdly sought to determine at what rates patients would accept and participate in MOSST. There were 2 drop-outs (16.7%), which was similar to other comparable studies in psychosis [31]. Reasons for dropping out were relapse and a patient’s decision that he or she did not need the treatment. The rate of acceptability and subjective impact of MOSST were adequate on all 10 parameters assessed. All patients who completed the full protocol rated the program as useful, generalizable to the real world, recommendable to others, and fun. For most participants, MOSST provided a challenge to identify and name their emotions, understand the limited influence of their expectations and desires on reality, and understand the mind of others and the existence of different points of view. Patients also rated positively the metacognitive approach of MOSST, and substantial improvements in therapist-patient communication over sessions were observed. In this regard, the use of self-disclosing and self-involving statements increased the intervention adherence.

The fourth aim was to examine the feasibility of the test battery and its administration. This proved efficient, particularly in ensuring there were no missing data. However, difficulties were encountered in ensuring consistency in PANSS scoring between assessors. For the RCT, additional documentation was developed and distributed to increase inter-rater reliability.

## Conclusions

Results collected from this pilot study are promising: both the methodology of the therapy protocol and data gathering seem adequate. This study, although a pilot in nature, is the first to suggest that Spanish outpatients with schizophrenia will accept a metacognitive SST, and shows evidence of improvements in psychosocial functioning. There are important limitations of the current study. Most notably, the sample size is insufficient and no control group was used. There is also a clear overrepresentation of males in the sample. Moreover, the duration of the treatment was brief, and results are needed from the ongoing trial to evaluate issues of dose and response. Finally, results from the ongoing trial are required to better understand whether changes in metacognition translate readily into improved daily functioning, clinical symptoms and outcomes in general.

## Abbreviations

BAI: Beck anxiety inventory
BDI–II: Beck depression inventory–II
ICD–10: International classification of diseases–tenth revision
MAI: Metacognition assessment interview
MAS–A: Metacognition assessment scale–abbreviated
MOSST: Metacognition-oriented social skills training
PANSS: Positive and negative syndrome scale
PSP: Personal and social performance scale
RCT: Randomized controlled trial
SST: Social skills training

## References

[1] Bellack AS, Morrison RL, Wixted JT, Mueser KT (1990). An analysis of social competence in schizophrenia. Br J Psychiatry.

[2] Dixon LB, Dickerson F, Bellack AS, Bennett M, Dickinson D, Goldberg RW (2010). The 2009 schizophrenia PORT psychosocial treatment recommendations and summary statements. Schizophr Bull.

[3] Almerie MQ, Okba Al Marhi M, Jawoosh M, Alsabbagh M, Matar HE, Maayan N, Bergman H. Social skills programmes for schizophrenia. Cochrane Database Syst Rev. 2015(6):Cd009006. 10.1002/14651858.CD009006.pub2 PMC7033904 26059249

[4] Pilling S, Bebbington P, Kuipers E, Garety P, Geddes J, Martindale B, et al. Psychological treatments in schizophrenia: II. Meta-analyses of randomized controlled trials of social skills training and cognitive remediation. Psychol Med. 2002;32(5):783–91. 10.1017/s0033291702005640 12171373

[5] Kurtz MM, Mueser KT (2008). A meta-analysis of controlled research on social skills training for schizophrenia. J Consult Clin Psychol.

[6] Granholm E, Holden J, Link PC, McQuaid JR, Jeste DV (2013). Randomized controlled trial of cognitive behavioral social skills training for older consumers with schizophrenia: defeatist performance attitudes and functional outcome. Am J Geriatr Psychiatry.

[7] Horan WP, Kern RS, Shokat-Fadai K, Sergi MJ, Wynn JK, Green MF (2009). Social cognitive skills training in schizophrenia: an initial efficacy study of stabilized outpatients. Schizophr Res.

[8] Kurtz MM, Richardson CL (2012). Social cognitive training for schizophrenia: a meta-analytic investigation of controlled research. Schizophr Bull.

[9] Lysaker PH, Erickson MA, Buck B, Buck KD, Olesek K, Grant ML (2011). Metacognition and social function in schizophrenia: associations over a period of five months. Cogn Neuropsychiatry.

[10] Inchausti F, Ortuño-Sierra J, Garcia-Poveda NV, Ballesteros-Prados A. Metacognitive abilities in adults with substance abuse treated in therapeutic community. Adicciones. 2016;29(2):74–82. 10.20882/adicciones.719 27749965

[11] Pinkham AE, Penn DL, Green MF, Buck B, Healey K, Harvey PD (2014). The social cognition psychometric evaluation study: results of the expert survey and RAND panel. Schizophr Bull.

[12] Vohs JL, Lysaker PH, Liffick E, Francis MM, Leonhardt BL, James A (2015). Metacognitive capacity as a predictor of insight in first-episode psychosis. J Nerv Ment Dis.

[13] James AV, Hasson-Ohayon I, Vohs J, Minor KS, Leonhardt BL, Buck KD (2016). Metacognition moderates the relationship between dysfunctional self-appraisal and social functioning in prolonged schizophrenia independent of psychopathology. Compr Psychiatry..

[14] Combs DR, Drake E, Basso MR, Lysaker PH, Dimaggio G, Brüne M (2014). An overview of social cognitive treatment interventions. Social cognition and Metacognition in schizophrenia.

[15] Ottavi P, D’Alia D, Lysaker P, Kent J, Popolo R, Salvatore G (2014). Metacognition-oriented social skills training for individuals with long-term schizophrenia: methodology and clinical illustration. Clin Psychol Psychother.

[16] Ottavi P, Pasinetti M, Popolo R, Salvatore G, Lysaker PH, Dimaggio G, Lysaker PH, Dimaggio G, Brüne M (2014). Metacognition-oriented social skills training. Social cognition and Metacognition in schizophrenia.

[17] Semerari A, Carcione A, Dimaggio G, Falcone M, Nicolò G, Procacci M (2003). How to evaluate metacognitive functioning in psychotherapy? The metacognition assessment scale and its applications. Clin Psychol Psychother.

[18] Safran JD, Muran JC (2000). Negotiating the therapeutic alliance. A relational treatment guide.

[19] Sturges JW (2012). Use of therapist self-disclosure and self-involving statements. Behav Ther.

[20] Katzow AW, Safran JD, Gilbert P, Leahy RL (2007). Recognizing and resolving ruptures in the therapeutic alliance. The therapeutic relationship in cognitive behavioral psychotherapies.

[21] Salvatore G, Dimaggio G, Popolo R, Lysaker PH (2008). Deficits in mindreading in stressful contexts and their relationships to social withdrawal in schizophrenia. Bull Menn Clin.

[22] de Jong S, van Donkersgoed RJ, Aleman A, van der Gaag M, Wunderink L, Arends J (2016). Practical implications of Metacognitively oriented psychotherapy in psychosis: findings from a pilot study. J Nerv Ment Dis.

[23] Apiquian R, Elena Ulloa R, Herrera-Estrella M, Moreno-Gomez A, Erosa S, Contreras V (2009). Validity of the Spanish version of the personal and social performance scale in schizophrenia. Schizophr Res.

[24] Lysaker PH, Vohs J, Hamm JA, Kukla M, Minor KS, de Jong S (2014). Deficits in metacognitive capacity distinguish patients with schizophrenia from those with prolonged medical adversity. J Psychiatr Res..

[25] Peralta V, Cuesta MJ (1994). Psychometric properties of the positive and negative syndrome scale (PANSS) in schizophrenia. Psychiatry Res.

[26] Beck AT, Steer RA, Brown GK (1996). BDI-II. Beck depression inventory second edition.

[27] Beck AT, Epstein N, Brown G, Steer RA (1988). An inventory for measuring clinical anxiety: psychometric properties. J Consult Clin Psychol.

[28] Arain M, Campbell MJ, Cooper CL, Lancaster GA (2010). What is a pilot or feasibility study? A review of current practice and editorial policy. BMC Med Res Methodol.

[29] Lysaker PH, Buck KD, Ringer JM (2007). The recovery of metacognitive capacity in schizophrenia across thirty two months of individual psychotherapy: a case study. Psychother Res.

[30] Lysaker PH, Davis LD, Eckert GJ, Strasburger A, Hunter N, Buck KD (2005). Changes in narrative structure and content in schizophrenia in long term individual psychotherapy: a single case study. Clin Psychol Psychother.

[31] Steel C, Hardy A, Smith B, Wykes T, Rose S, Enright S, Hardcastle M, Landau S, Baksh MF, Gottlieb JD et al. Cognitive-behaviour therapy for post-traumatic stress in schizophrenia. A randomized controlled trial. Psychol Med. 2017;47(1):43–51. 10.1017/S0033291716002117 27650432

